# Strengthening theoretical assessment design in nursing education: Advancing SDG 4

**DOI:** 10.4102/hsag.v30i0.3061

**Published:** 2025-08-21

**Authors:** Gabieba Donough, Katlego Mthimunye, Felicity Daniels

**Affiliations:** 1 Faculty of Community and Health Sciences, University of the Western Cape, Cape Town, South Africa

**Keywords:** Bloom’s Taxonomy, competency-based assessment, moderation, nursing education, theoretical assessment, SDG 4

## Abstract

**Background:**

Quality theoretical assessments in nursing education are essential for achieving Sustainable Development Goal 4 (ensuring inclusive and equitable quality education and promoting lifelong learning opportunities for all). However, misalignment with National Qualification Framework (NQF) standards, an overreliance on recall-based questions and inadequate educator training undermine the fairness of assessments and students’ ability to demonstrate clinical competence.

**Aim:**

This study explores theoretical assessment design in nursing education, evaluating its alignment with NQF standards, competency-based assessments and Sustainable Development Goal 4.

**Setting:**

Conducted at a South African nursing school offering undergraduate nursing programmes.

**Methods:**

A multimethod approach integrated educator interviews, document reviews of moderation reports, a scoping review and a student survey to investigate assessment practices.

**Results:**

While educators valued Bloom’s Taxonomy, over-reliance on recall-based questions limited critical thinking, clinical decision-making and competency development. Misalignment with NQF standards caused inconsistencies in cognitive demand, while gaps in moderation processes impacted assessment validity and fairness. Educators faced challenges because of limited training and support, and students struggled with ambiguous and linguistically complex assessments that hindered their ability to demonstrate competency.

**Conclusion:**

Strengthening assessment design, moderation and educator training is essential to improving competency-based assessment practices in nursing education. Policy reforms promoting fair, transparent, and competency-driven assessments will enhance graduate preparedness, ensure alignment with NQF standards and support SDG 4’s goal of quality education.

**Contribution:**

This study provides empirical evidence supporting assessment policy improvements, promoting structured, competency-based assessments that enhance fairness, deepen learning and align with nursing education standards.

## Introduction

Quality nursing education is essential for developing clinical competency, ensuring that graduates can make informed decisions that directly impact patient care and safety (Falcó-Pegueroles et al. 2021). Theoretical assessments are crucial in evaluating students’ knowledge application, critical thinking and decision-making skills, which are fundamental to professional readiness in clinical settings (SAQA 2022). However, current assessment practices often rely on recall-based questions, limiting students’ ability to engage in higher-order cognitive processes such as clinical reasoning and decision-making (Dos Reis et al. 2022; Fayilane 2017). Studies indicate that misaligned and poorly designed assessments contribute to inconsistent competency outcomes among nursing graduates, which can have long-term effects on healthcare delivery and patient outcomes (Donough 2023; Dos Reis et al. 2022; Ilhami 2024). Addressing these challenges through fair, competency-based and transparent assessment practices is crucial to achieving Sustainable Development Goal 4 (SDG 4), which advocates for quality education and lifelong learning opportunities (Sorooshian 2024).

Despite the importance of competency-based assessments, research highlights a gap in alignment between assessment practices and National Qualification Framework (NQF) standards, leading to unfair and unreliable assessments (Mokwele & Chetty 2022; SAQA 2022). While these studies emphasise the need for higher-order cognitive engagement, many assessments still over-rely on rote memorisation, failing to measure students’ readiness for clinical environments adequately (Falcó-Pegueroles et al. 2021). In addition, moderation processes are inconsistently implemented, resulting in disparities in assessment fairness across institutions (Hecker et al. 2024). This study fills this gap by exploring how theoretical assessments in nursing education are designed, their impact on student learning and their alignment with SDG 4 and competency-based assessment practices.

This study is grounded in constructive alignment theory (Biggs 1996) and Bloom’s Taxonomy (Bloom 1956), both essential for structuring competency-based assessments. Constructive alignment theory posits that assessments should be intentionally aligned with learning outcomes and teaching strategies to ensure meaningful learning. In nursing education, misaligned assessments hinder the transition from theoretical knowledge to clinical competency, affecting graduate readiness for professional practice. Bloom’s Taxonomy provides a framework for categorising cognitive learning objectives, emphasising the progression from basic recall to higher-order thinking skills, which are critical for nursing students to develop clinical reasoning, problem-solving abilities and clinical competency. Competency-based assessments are designed to develop clinical reasoning and decision-making skills rather than simply testing rote memorisation (Falcó-Pegueroles et al. 2021). Integrating these frameworks allows for a structured analysis of assessment design, cognitive demand and alignment with competency-based assessment practices to support SDG 4’s commitment to quality education and improve graduate clinical readiness.

### Aim

This study explores theoretical assessment design in nursing education, evaluating its alignment with NQF standards, competency-based assessments and Sustainable Development Goal 4.

## Research methods and design

This study employed a multimethod research design, integrating both qualitative and quantitative approaches for data collection, analysis and interpretation (Creswell & Creswell 2017). This approach ensured methodological rigour, enhancing the validity and reliability of findings while providing a comprehensive understanding of theoretical assessment practices in nursing education.

### Setting

The study was conducted at a South African nursing school, focusing on undergraduate students enrolled in the Bachelor of Nursing (BN) programme. This programme consists of two tracks: the 4-year mainstream BN programme and the 5-year Extended Curriculum Programme (ECP). The academic year levels are structured according to the NQF standards, ensuring competency progression aligned with national educational policies. Students were categorised as follows:

NQF Level 5: First-year students in ECP/BN1

NQF Level 6: Second-year students (BN2)

NQF Level 7: Third-year students (BN3)

NQF Level 8: Fourth-year students (BN4)

### Study population and sampling

The study population included nursing educators, moderation reports, literature on theoretical assessment design and nursing students.

Nursing educators were purposively selected based on their expertise in assessment design, undergraduate teaching and curriculum development. To ensure representation across different academic levels, educators from each NQF level were included. In-depth interviews continued until data saturation was reached, resulting in nine nursing educators participating in in-depth interviews (Braun & Clarke 2021). This approach ensured a deeper understanding of assessment practices across all levels of undergraduate nursing education.

For the document review, internal and external moderation reports from the Bachelor of Nursing R425 undergraduate programme (2015–2019) were purposively sampled for relevance to assessment alignment and fairness. This timeframe aligned with the final intake of students for the R425 programme before its national phase-out in 2019 (SANC 2021). Only nursing modules with completed final theoretical assessment moderation were considered, and reports had to provide assessment feedback on alignment, fairness and competency to meet inclusion criteria. Incomplete reports and clinical assessment reports were excluded. Of the 150 collected reports covering 18 nursing modules, 70 met the inclusion criteria, comprising 22 internal and 48 external moderation reports.

A scoping review was conducted to explore the literature on theoretical assessment design by educators, following PRISMA guidelines to ensure transparency and rigour. The review was guided by Arksey and O’Malley (2005) five-stage framework, which included the following stages:

1. Determining the following research question: ‘what are the literature on theoretical assessment practices by educators’ to define the focus of the review.
2. Identifying relevant studies by conducting a systematic search to find articles related to the research question. Databases such as Education Resource Information Centre (ERIC), Ebsco Host and Cumulative Index of Nursing and Allied Health Literature (CINAHL) were accessed through library services.
3. Performing study selection through screening studies based on predefined inclusion criteria, that is, studies on theoretical assessment design by educators.
4. Charting the data by extracting key information from the selected studies. A systematic search initially retrieved 1015 articles, which were subsequently narrowed down to 12 relevant studies from 11 international countries.
5. Collating, summarising and reporting the results by analysing and synthesising the findings to highlight trends and gaps in the literature.

A survey was conducted among 302 undergraduate nursing students across all academic levels, achieving a 100% response rate. The participants were selected based on their experience with writing final exams, as this was crucial for gathering relevant insights into their assessment experiences. Because the final exams undergo moderation, including only students who had written them ensured a more accurate evaluation of assessment practices. Postgraduate students were excluded to maintain focus on the undergraduate assessment experience. Participants were from the following academic levels: ECP/BN1 (*n* = 77), BN2 (*n* = 75), BN3 (*n* = 68) and BN4 (*n* = 82).

### Data collection

After ethical approval, a pretesting phase was conducted for each data source to assess the clarity of research questions, the effectiveness of data collection tools and the feasibility of data management procedures. Based on insights gained from the pretesting, modifications were made to the instruments to enhance accuracy, reliability and consistency in data collection.

In-depth interviews were conducted with nine purposively selected nursing educators to explore their experiences with theoretical assessment design. Data were collected using semi-structured interviews, which provided flexibility while ensuring consistency across participants. All interviews were audio recorded, transcribed verbatim and thematically analysed to identify recurring patterns and themes related to assessment alignment, fairness and competency development.

A document review was conducted to examine internal and external moderation reports of final theoretical assessments. Moderation reports from 2015 to 2019 were obtained in both electronic and hard copy formats through the administrator from the selected institution under study. These reports were reviewed to assess alignment with learning outcomes, cognitive demand and quality assurance practices in nursing education.

A scoping review was conducted, and a three-stage screening process was followed by using data extraction tools. The Title Reading and Extraction Tool (TRET), Donough, 2023, identified relevant articles based on titles. The Abstract Reading and Extraction Tool (ARET) further refined the selection, retaining the articles for full-text screening. The data extraction tool (DET) facilitated a detailed analysis of study type, participants, instruments and findings. A manual search of reference lists yielded additional sources, leading to a final inclusion of 12 relevant articles. The studies originated from 11 countries: Australia (*n* = 1), Canada (*n* = 1), Chile (*n* = 1), Iran (*n* = 1), the Kingdom of Saudi Arabia (KSA) (*n* = 1), New Zealand (*n* = 1), Norway (*n* = 1), Spain (*n* = 1), Sudan (*n* = 1), the United Kingdom (*n* = 1) and the United States (*n* = 2) (Donough, Daniels & Mthimunye 2022).

The student survey used the Assessment Experience Questionnaire (AEQ) 5.1, originally developed by Gibbs and Simpson (2003) and later revised by Batten, Jessop and Birch (2019). This standardised questionnaire, designed to assess student experiences of assessments within their academic programme, employs a five-point Likert scale ranging from strongly agree to strongly disagree. Permission to use the AEQ for this study was obtained from the respective authors. The questionnaire is structured into two sections: Section A collects demographic data through six closed-ended questions, while Section B focuses on students’ experiences with assessments, using Likert-scale statements to capture their perspectives. The AEQ has demonstrated high reliability, with an original Cronbach’s alpha coefficient exceeding 0.80 (Batten et al. 2019), ensuring the consistency and validity of responses.

### Data analysis

Thematic analysis was applied to qualitative data, identifying recurring patterns related to assessment alignment, fairness and competency development (Braun & Clarke 2021). Descriptive statistics summarised student survey responses, detailing demographic variables and assessment experiences. Inferential statistics included the Kruskal–Wallis test to examine differences in student experiences across academic levels, and the Pearson’s Chi-square test to assess group differences related to assessment challenges. These analyses ensured statistical rigour and validity.

### Triangulated results

To strengthen the validity and reliability of the findings, triangulation was employed by integrating multiple data sources (Kawar et al. 2024): educator in-depth interviews, moderation reports, the scoping review and student survey. This approach provided a comprehensive interpretation of theoretical assessment practices in nursing education by capturing diverse perspectives. Synthesising these qualitative and quantitative insights offered a deeper understanding of assessment challenges and highlighted the urgent need for structured assessment reforms aligned with SDG 4 and competency-based assessments.

### Measures of trustworthiness, reliability and validity

This study ensured rigour through established principles of trustworthiness for qualitative data and reliability and validity for quantitative components (Creswell & Creswell 2017). Data triangulation across educator interviews, moderation reports, the scoping review and the student survey enhanced credibility and strengthened methodological robustness.

Trustworthiness was established by enhancing credibility through triangulation and member checking, enabling participants to verify transcripts and interpretations. Transferability was supported by providing detailed descriptions of the study context and participant selection. Dependability was ensured through an audit trail documenting key research decisions, while confirmability was maintained through researcher reflexivity to limit bias.

For the quantitative data, pilot testing refined the student survey’s clarity and content validity. Internal consistency was confirmed using Cronbach’s alpha, and construct validity was established by aligning survey items with existing literature. Statistical tests, including the Kruskal–Wallis and Pearson’s Chi-square, ensured analytical rigour and precision.

Together, these strategies reinforced the study’s credibility, dependability and relevance, ensuring robust findings that inform quality learning and support SDG 4 in nursing education.

### Ethical considerations

Ethical approval was obtained from the Human Social Sciences Research Ethics Committee (Reference: HS20/8/19). Institutional permission was granted, and all participants provided written consent (Creswell & Creswell 2017). Data were anonymised and securely stored according to institutional guidelines.

## Results

The study employed a multimethod approach, integrating qualitative and quantitative data to comprehensively explore theoretical assessments in nursing education. Insights were drawn from in-depth interviews with educators, moderation reports, a scoping review of literature and a student survey, each providing a distinct perspective on quality theoretical assessments. By triangulating these findings, a holistic analysis was achieved, ensuring a nuanced interpretation of assessment design. Four themes emerged from different data sources. To facilitate interpretation, Table 1 systematically presents how each data source contributed to the themes identified.

**TABLE 1:** Triangulated findings on assessment practices in nursing education.

Themes	Findings from in-depth interviews (educators)	Findings from document review (moderation reports)	Findings from scoping review (literature)	Findings from student survey
The Role of Bloom’s Taxonomy in Effective Assessment Design	Educators use Bloom’s Taxonomy in assessment design but lean heavily on lower-order questions. ‘I look at things like Bloom’s Taxonomy … formulate my exam papers.’ (Interview, Educator, Participant 1)	Moderation reports highlight the limited cognitive complexity in assessments. ‘Level of questions is primarily based on understanding.’ (3rd Year, External Moderator, Moderator A)	Literature confirms that educators rely on Bloom’s levels when designing assessments, but overuse of lower-order questions limits critical thinking (Abdalla 2013; Killingsworth, Kimble and Sudia 2015).	Students did not reference Bloom’s Taxonomy directly, but older students engaged in deeper learning, suggesting links between assessment design and cognitive engagement.
Alignment with Learning Outcomes, NQF Standards, and Quality Assurance	Educators align assessments with NQF Levels. ‘Your NQF level … you also need to take into consideration when designing assessments.’ (Interview, Educator, Participant 8)	Moderation reports reveal gaps in effective NQF alignment and Learning Outcomes. ‘Each paper lacked assessment of all specific outcomes for this module.’ (2nd Year, Internal Moderator, Moderator C)	Literature stresses that assessments should be constructively aligned to ensure fairness and uphold academic standards (Killingsworth et al. 2015).	Students did not explicitly reference NQF alignment but found variability in assessment difficulty, suggesting inconsistencies in cognitive demand, implying variable alignment with outcomes.
Challenges in Assessment Design and Educator Preparedness	Educators report time constraints and pressure in assessment design. ‘… the one thing that is sometimes difficult, is that you’re always under such a lot of pressure in setting your papers ….’ (Interview, Educator, Participant 8)	Moderation reports highlight marking misalignment because of unclear assessment structuring. ‘If you really want the students to discuss this issue, four marks are not enough.’ (3rd Year, External Moderator, Moderator B)	Literature states that many educators struggle with assessment design because of a lack of clear guidelines and formal training (Killingsworth et al. 2015; Meyer et al. 2010; Norton, Norton & Shannon 2013).	Students did not explicitly discuss educator preparedness but expressed concerns over inconsistent, linking to assessment quality.
Language Barriers and Technical Challenges in Assessment Design	Educators acknowledge language difficulties for both themselves and students. ‘Language plays a big role I have a better understanding of many things in my own mother tongue.’ (Interview, Educator, Participant 7)	Moderation reports identify language clarity and technical issues, such as poorly worded questions. ‘Typing errors, questions that need rewording/reconstruction.’ (3rd Year, Internal Moderator, Moderator C)	Literature indicates that educators face difficulties in designing linguistically appropriate assessments, with comfort in assessment design improving with experience (Norton et al. 2013).	Survey results show 59.3% of students’ first language is not English, with difficulties understanding ambiguous questions.

Note: Please see full reference list of this article: Donough, G., Mthimunye, K. & Daniels, F., 2025, ‘Strengthening theoretical assessment design in nursing education: Advancing SDG 4’, *Health SA Gesondheid* 30(0), a3061. https://doi.org/10.4102/hsag.v30i0.3061 for more information.

NQF, National Qualification Framework.

### The role of Bloom’s taxonomy in effective assessment design

The finding from in-depth interviews with nurse educators revealed that while Bloom’s Taxonomy is used to scaffold assessments, there is a strong reliance on lower-order cognitive domains such as recall and comprehension. This approach limits students’ development of critical thinking and clinical reasoning skills essential for practice readiness. Moderation reports confirmed that assessments predominantly test lower-order thinking, with comments such as ‘Level of questions is primarily based on understanding’. The scoping review of literature corroborated these findings, highlighting a trend where assessments emphasise memorisation over critical engagement (Abdalla 2013; Killingsworth et al. 2015). Although students did not explicitly reference Bloom’s Taxonomy, survey results showed that older students demonstrated deeper engagement with assessment preparation, suggesting a link between cognitive maturity and learning approach. These findings highlight the need for a more balanced use of Bloom’s levels, with greater emphasis on higher-order thinking to support competency development.

### Alignment with learning outcomes, National Qualification Framework standards and quality assurance

Interviews with educators demonstrated awareness of the need to align assessments with NQF levels and learning outcomes, as illustrated by one educator stating, ‘Your NQF level … you also need to take into consideration when designing assessments’. However, document reviews of moderation reports identified inconsistencies, such as a lack of assessment for all specific outcomes within modules, leading to disparities in cognitive demand and fairness. The scoping review reinforced the importance of constructive alignment to uphold assessment standards (Killingsworth et al. 2015). Although students did not directly reference NQF alignment, variability in assessment difficulty reported in the survey indicates inconsistencies in cognitive expectations. These findings point to the need for standardised, competency-aligned assessment frameworks and robust moderation processes to ensure fairness and quality assurance in nursing education.

### Challenges in assessment design and educator preparedness

Educators reported time constraints, workload pressures and insufficient training as major barriers to effective assessment design, with one noting, ‘… the one thing that is sometimes difficult is that you’re always under such a lot of pressure in setting your papers …’. Moderation reports echoed these challenges, highlighting issues such as unclear question structures and marking inconsistencies: ‘If you really want the students to discuss this issue, four marks are not enough’. The scoping review highlighted the need for clear guidelines and training to build assessment capacity among educators (Killingsworth et al. 2015; Meyer et al. 2010). Student survey findings, while not directly referencing educator preparedness, reflected these gaps through reports of inconsistent marking and unclear instructions. These results collectively highlight the need for structured professional development and support systems to enhance educators’ ability to design fair, competency-based assessments.

### Language barriers and technical issues in assessment design

Language-related challenges were evident in educator interviews, with one participant observing, ‘Language plays a big role … I have a better understanding of many things in my own mother tongue’. Moderation reports identified similar issues, pointing to unclear phrasing, typographical errors and questions requiring rewording. The scoping review supports the importance of clear, accessible language in assessments, particularly in multilingual contexts (Norton et al. 2013). Student survey data revealed that 59.3% of respondents’ first language is not English, and many reported struggling with unclear or ambiguous questions, which may hinder their ability to demonstrate knowledge and apply critical thinking in clinical scenarios. These findings highlight the need for assessments to be written in clear, simple and straightforward language that is free from ambiguity and complex phrasing.

## Discussion

This study highlights critical challenges in theoretical assessment design in nursing education, including over-reliance on lower-order cognitive assessments, misalignment with national standards and moderation, inadequate educator training and language barriers. The triangulation of data sources, including educator interviews, moderation reports, the scoping review and the student survey, revealed systemic gaps in quality theoretical assessment practices that undermine the goals of SDG 4 (Sorooshian 2024): equitable, quality education that equips students with the knowledge, skills and critical thinking required for professional nursing practice.

One of the main problems identified is the over-reliance on lower-order cognitive questions in theoretical assessments. This study highlights how theoretical assessment design practices, while informed by a taxonomy like Bloom’s Taxonomy, remain constrained by an over-reliance on lower-order questions. As a result, assessments risk measuring rote knowledge rather than clinical application, undermining the goals of competency-based assessments and limiting students’ ability to demonstrate readiness for professional practice. Tai, Ajjawi and Umarova (2024) and Boyer and Chickering (2024) explained that competency-based assessments in nursing require assessments that foster higher-order cognitive skills, such as clinical reasoning, problem solving and decision-making. Without intentional design that scaffolds learning beyond recall and comprehension, students may develop fragmented knowledge rather than integrated competencies essential for safe patient care (Biggs, Tang & Kennedy 2022; Ergashevich 2024; Govindasamy & Kwe 2020). Strengthening theoretical assessment design to integrate competency-based assessments ensures graduates can apply critical thinking and clinical reasoning in professional practice. Advancing structured, competency-based assessments in nursing education supports SDG 4 by fostering equitable, quality education that prepares students for complex healthcare challenges.

Another critical issue in theoretical assessment design is the misalignment with NQF standards and intended learning outcomes. While constructive alignment is widely recognised in educational literature as a key principle for ensuring fair and meaningful assessment (Biggs & Tang 2015; Hecker et al. 2024), this study revealed that theoretical assessment design often fails to adequately reflect expected cognitive levels and competencies. Misalignment results in disparities in cognitive demand, which either under-challenges students or sets unrealistic expectations for demonstrating clinical reasoning and problem-solving abilities. This variability compromises the fairness and reliability of competency-based assessments, contributing to inconsistent graduate outcomes across institutions (Hecker et al. 2024; Irenka & Ireland 2021; Villarroel et al. 2024). Without aligning the NQF standards and expected outcomes, students’ risk being inadequately prepared for clinical practice. Educators must prioritise alignment between learning outcomes, NQF standards and assessments to ensure that nursing graduates achieve the competencies necessary for professional readiness. Ensuring that the principles of assessments are maintained and implementing clear policies can reinforce fairness, transparency and effective assessment practices that support the goals of SDG 4 for quality, equitable education.

A persistent challenge in nursing education is the lack of educator preparedness in designing quality assessments. Despite the critical role of assessment in shaping learning outcomes, many nursing educators face significant barriers, including workload pressures, limited time for quality assessment design and insufficient training in assessment literacy (Abou Hashish, Alnajjar & Rawas 2025; Griffiths et al. 2020). This study revealed that without structured training and institutional support, educators may rely on traditional, lower-order questions that fail to assess critical thinking, clinical reasoning or professional decision-making abilities. Structured training programmes are essential for equipping educators with the skills to design robust, quality assessments that reflect real-world clinical demands (Chizengo-Thawani & Shawa 2022; Villarroel et al. 2024). Addressing these gaps is crucial for ensuring that theoretical assessments accurately measure the competencies necessary for safe and effective nursing practice. Higher education institutions (HEIs) must invest in ongoing professional development, mentorship programmes and curriculum-aligned assessment workshops to empower educators in designing fair, transparent and competency-based assessments (Chukwu et al. 2024). Strengthening educator capacity is integral to advancing competency-based assessments in nursing education and aligning with SDG 4’s call for inclusive, equitable and quality education for all.

Language and technical barriers in theoretical assessment design present a significant obstacle to fair and accurate quality assessment in nursing education. In multilingual contexts such as South Africa (Khoza-Shangase & Kalenga 2024), where English is the primary language of instruction at the institution in this study, students learning in a language other than their mother tongue may struggle to comprehend complex or ambiguously worded assessment questions. This study highlights how language challenges not only impact students’ theoretical assessment performance but also hinder their ability to apply knowledge in clinical settings, where accurate interpretation and communication are critical for patient safety. Ambiguous phrasing, typographical errors and unclear instructions further exacerbate these issues, creating inequities in student performance and disadvantaging those who require clearer language scaffolding (Arslan et al. 2024; Oliinyk et al. 2024). Arslan et al. (2024) and Bostrom (2020) highlight the importance of culturally responsive and linguistically inclusive assessments (that is clear and simple) in supporting equitable learning and competency demonstration. Institutions must implement structured assessment review processes to ensure clarity, inclusivity and accessibility of theoretical assessments. Addressing language barriers is not only a matter of fairness but also a crucial step in enhancing the validity and reliability of quality assessments. Strengthening linguistically inclusive assessment design will support students’ ability to demonstrate clinical reasoning and critical thinking skills, contributing to the achievement of SDG 4 and equitable, quality education in nursing.

### Limitation

While this study provides valuable insights into theoretical assessment practices in nursing education, its findings are context specific and reflect practices within a single institution. Future research should conduct larger-scale, cross-institutional comparisons to validate and expand upon these findings, ensuring greater relevance and applicability to diverse nursing education systems.

### Recommendations

- Institutions should establish ongoing professional development programmes to equip educators with the skills to design fair, transparent and competency-based assessments that promote higher-order thinking, clinical reasoning and decision-making.
- Institutions must establish clear, standardised moderation frameworks that ensure assessments align with NQF standards, reduce disparities in cognitive demand and uphold fairness and reliability across academic levels.
- Institutions should integrate a structured technical review process to ensure assessment questions are clear, accessible and free of language barriers that may disadvantage students from diverse linguistic backgrounds.

These recommendations will enhance the quality and fairness of theoretical assessment design in nursing education, strengthen educator capacity, and ensure that nursing graduates are well prepared to meet professional standards and contribute meaningfully to the healthcare system in alignment with SDG 4.

## Conclusion

This study offers comprehensive insights into the challenges of designing theoretical assessments in nursing education. It highlights the need for fair, transparent and quality theoretical assessment design in nursing education to advance SDG 4 and professional competency development. Findings reveal that while Bloom’s Taxonomy is valuable for designing theoretical assessments, excessive reliance on recall-based questions limits critical thinking and clinical reasoning. In addition, misalignment with NQF standards, variability in the application of moderation feedback, inadequate educator training and language barriers compromise assessment fairness and student preparedness for clinical practice. Although moderation processes are intended to improve assessment quality, the findings indicate that their effectiveness depends on how consistently feedback is implemented and how well moderation practices are standardised. Strengthening educator training in quality theoretical assessment design, reinforcing effective moderation practices and ensuring accessible and well-structured assessments are essential steps towards reform. By aligning assessment frameworks with education standards, HEIs can enhance assessment reliability, graduate readiness and equitable learning, ensuring that nursing graduates are well equipped for clinical practice and lifelong professional growth.
